# Transcriptome and Difference Analysis of Fenpropathrin Resistant Predatory Mite, *Neoseiulus barkeri* (Hughes)

**DOI:** 10.3390/ijms17060704

**Published:** 2016-05-27

**Authors:** Lin Cong, Fei Chen, Shijiang Yu, Lili Ding, Juan Yang, Ren Luo, Huixia Tian, Hongjun Li, Haoqiang Liu, Chun Ran

**Affiliations:** 1 Citrus Research Institute, Southwest University/Chinese Academy of Agricultural Sciences, Chongqing 400712, China; conglin@cric.cn (L.C.); yushijiang@sinocitrus.com (S.Y.); dinglijiayoua@126.com (L.D.); yangjuan@sinocitrus.com (J.Y.); koalox@163.com (R.L.); huixia88_1012@163.com (H.T.); lihongjun@cric.cn (H.Li.); liuhaoqiang@cric.cn (H.Liu); 2 Sinofert Holdings Limited, Henan Branch, Zhengzhou 450000, China; chenfei6034@163.com

**Keywords:** *Neoseiulus barkeri*, transcriptome, fenpropathrin resistant, VGSC, P450, GST

## Abstract

Several fenpropathrin-resistant predatory mites have been reported. However, the molecular mechanism of the resistance remains unknown. In the present study, the *Neoseiulus barkeri* (*N. barkeri*) transcriptome was generated using the Illumina sequencing platform, 34,211 unigenes were obtained, and 15,987 were manually annotated. After manual annotation, attentions were attracted to resistance-related genes, such as voltage-gated sodium channel (VGSC), cytochrome P450s (P450s), and glutathione S-transferases (GSTs). A polymorphism analysis detected two point mutations (E1233G and S1282G) in the linker region between VGSC domain II and III. In addition, 43 putative P450 genes and 10 putative GST genes were identified from the transcriptome. Among them, two P450 genes, *NbCYP4EV2* and *NbCYP4EZ1*, and four GST genes, *NbGSTd01*, *NbGSTd02*, *NbGSTd03* and *NbGSTm03*, were remarkably overexpressed 3.64–46.69-fold in the fenpropathrin resistant strain compared to that in the susceptible strain. These results suggest that fenpropathrin resistance in *N. barkeri* is a complex biological process involving many genetic changes and provide new insight into the *N. barkeri* resistance mechanism.

## 1. Introduction

*Neoseiulus barkeri* (Hughes) (*N. barkeri*) is a polyphagous predatory mite [1] that feeds on different prey, such as tetranychid mites [2], tarsonemid mites [3], eriophyid mites [4], and thrips [5]. Due to its irreplaceable biological traits, such as a wide distribution, polyphagy, short life span, ease of culture, high fecundity, and mobility, *N. barkeri* is now regarded as one of the most valuable and successful commercial biological control agents for thrips and spider mites [6,7,8,9,10]. In particular, *N. barkeri* has served as a native commercialized predatory pest control mite in many cropping systems for several years in China [11].

Considering the complexity of pest species and their population dynamics, pest management heavily relies on a wide spectrum of chemicals, such as pyrethroids, which inevitably cause mass mortality of natural enemies. Thus, screening for chemically-resistant predatory mites is a practical way to balance pesticide and biological control by reducing the side-effects of chemical agents. Ten chemically-resistant predatory mites have been reported [12,13,14] and successfully applied to pest control [7,15,16,17]. Unfortunately, little effort has been paid to explore the resistance mechanisms in these mites, which highly limits enhancement of this strategy. However, studies on the chemical-resistance mechanisms in insects and leaf mites have achieved important breakthroughs. Two major mechanisms are involved in pyrethroid-resistance, such as enhanced detoxification metabolism by cytochrome P450s (P450s), glutathione S-transferases (GSTs), and carboxylesterases, as well as reduced sensitivity to pyrethroid-targeted proteins, such as voltage-gated sodium channels (VGSCs) via mutations [18].

Studies on genetic mechanisms have entered a new era since the emergence of transcriptome sequencing technologies, and transcriptome analysis has been effectively used to identify resistance-related genes in many insects [19,20,21,22,23]. However, this technology has not been extensively explored in acarology, as only *Metaseiulus occidentalis* [24] and *Phytoseiulus persimilis* [25] have been sequenced and reported, but neither is relevant to chemical resistance.

In this study, we illustrate the molecular resistance mechanism of *N. barkeri* against fenpropathrin by sequencing the *N. barkeri* transcriptome using Illumina technology. After annotation, we compared variations in sequence and expression of VGSC, P450, and GST genes between *N. barkeri* resistant and susceptible strains. RNA-sequencing, sequence polymorphism, and quantitative analyses were used to provide new insight into chemical resistance in predatory mites.

## 2. Results

### 2.1. Resistance Selection

The resistance level of *N. barkeri* against fenpropathrin was represented by mortality. The bioassay results indicated that a fenpropathrin-resistant strain of *N. barkeri* was obtained (Table 1). The lethal concentration 50 (LC_50_) increased from 35.79 to 22,190.91 mg/L, with a resistance ratio of 619.96-fold, compared to that of the susceptible strain.

### 2.2. RNA-Sequencing Analysis

A cDNA library was constructed from a pooled RNA sample isolated from eggs, larva, nymphs, and adults of equal ratios to gain a more comprehensive understanding of the gene expression pattern in the fenpropathrin resistant *N. barkeri* strain. In total, 25,192,607 total reads and 5,087,856,659 nucleotides (nt) were obtained from the transcriptome data and further assembled into 1,385,792 contigs, 50,462 transcripts, and 34,211 unigenes (Table 2). The length distributions (Figure S1, Supplementary Material) demonstrated that most of the contigs (98.00%) and the majority of transcripts (58.22%) were 0–300 and 200–1000 bp in length, respectively, and that 37.03% of the unigenes were 500–3000 bp.

### 2.3. Annotation of Predicted Proteins

All distinct unigenes were subjected to a BLASTX search against the nonredundant (nr) database at the National Center for Biotechnology Information (NCBI), as well as the Swiss-Prot, Kyoto Encyclopedia of Genes and Genomes (KEGG), Cluster of Orthologous Groups (COG), and Gene Ontology (GO) databases to be annotated. In total, 15,987 (46.73%) of all 34,211 unigenes were matched successfully to known genes (Table 2), with 15,866 from the nr database, 10,486 from Swiss-Prot, 5445 from KEGG, 5673 from COG, and 8707 from the GO databases. The *E*-value distribution of the annotated unigenes suggested that 63.63% of the mapped unigenes had very significant homology (<*E*^−50^); however, 36.37% of the mapped unigenes ranged between *E*^−50^ and *E*^−5^ (Figure S2A, Supplementary Material). The species distribution further indicated that 12,033 unigenes (75.99%) had their best hits with sequences from *M. occidentalis*, followed by those from *Ixodes scapularis* (2.76%), *Acyrthosiphon pisum* (0.80%), *Branchiostoma floridae* (0.66%), *Daphnia pulex* (0.63%), *Capitella teleta* (0.63%), and other species (18.53%) (Figure S2B, Supplementary Material).

### 2.4. Functional Annotation

According to the results, 8707 unigenes were classified into 73,709 GO terms (40,628 in biological process, 21,967 in cellular component and 11,114 in molecular function) and 48 subcategories (21 in biological process, 14 in cellular component and 13 molecular function) (Figure S3, Supplementary Material). Of the biological process, “cellular process” (5655 GO terms), “metabolic process” (5255 GO terms), and “developmental processes” (4005 GO terms) were the most abundant. Of the cellular component, “synapse” (4854 GO terms), “ell” (4627 GO terms), and “organelle” (3675 GO terms) were most represented. “Binding” (4454 GO terms) and “catalytic activity” (4304 GO terms) were the top two molecular function subcategories. In total, 5673 unigenes were mapped and sorted into 25 COG categories. Among them, “general function prediction” (1702, 30.00%); “replication, recombination, and repair” (645, 11.37%); “translation, ribosomal structure, and biogenesis” (600, 10.58%); and “transcription” (525, 9.25%) were most represented. Seven and four unigenes were found in the categories “cell motility” and “nuclear structure”, respectively. No unigene was found in the “extracellular structures” category (Figure S4, Supplementary Material).

A total of 5,445 unigenes (34.06%) were mapped to 217 metabolic pathways by the KEGG analysis. The most abundant KEGG pathways were: “ribosome” (251, 4.61%), “protein processing in endoplasmic reticulum” (211, 3.88%), “RNA transport” (182, 3.34%), “oxidative phosphorylation” (162, 2.98%), and “lysosome” (160, 2.94%) (Figure S5, Supplementary Material).

### 2.5. Cloning of the N. barkeri Sodium Channel Gene and Verification of Point Mutations

**Figure 1:**
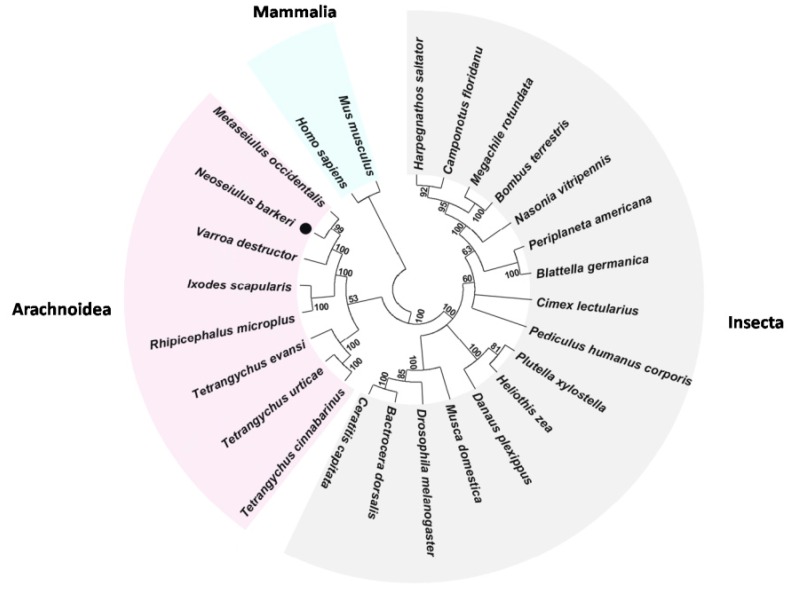
Phylogenetic analysis of sodium channels. The amino acid sequences generated from *N. barkeri* and 25 other species were selected to construct the phylogenetic tree using the Neighbor-joining method with a bootstrap value of 1000. The dot represents the protein sequence of the *N. barkeri* sodium channel.

Eight cDNA fragments of 546–1180 bp were obtained and finally assembled. The complete open reading frame (ORF) of the *N. barkeri* sodium channel (*NbSc*, GenBank accession number: KT768110) was 6175 bp, encoding a 2058 amino acid (aa) polypeptide, with a calculated molecular mass of 233.75 kDa and an isoelectric point of 5.09. The alignment indicated that the deduced *Nb*Sc aa sequence shared 91.04%, 80.48%, 69.00%, 55.09%, and 41.98% identities with the sodium channels from *M. occidentalis* (XP_003741737), *Varroa destructor* (AAP13992), *I. scapularis* (XM_002407075), *Drosophila melanogaster* (AAB59195), and *Homo sapiens* (NP_066287), respectively. In addition, similar to other sodium channels, *Nb*Sc also harbored four homologous domains (I–IV) with six transmembrane segments (S1–S6) in each domain (Figure S6, Supplementary Material). The topology supported that *Nb*Sc has a closer evolutionary relationship with *M. occidentalis*, which clustered into a single clade from other arachnoid species and insects (Figure 1). A glutamic acid to glycine replacement at aa 1233 (*N. barkeri* numbering) and a serine to glycine replacement at aa 1282 (*N. barkeri* numbering) were located in the linker region between domain II and III (1172 and 1210 *Musca domestica* numbering, GenBank accession number: AAB47604).

### 2.6. Identification and Differential Expression Analysis of P450s and GSTs

**Figure 2:**
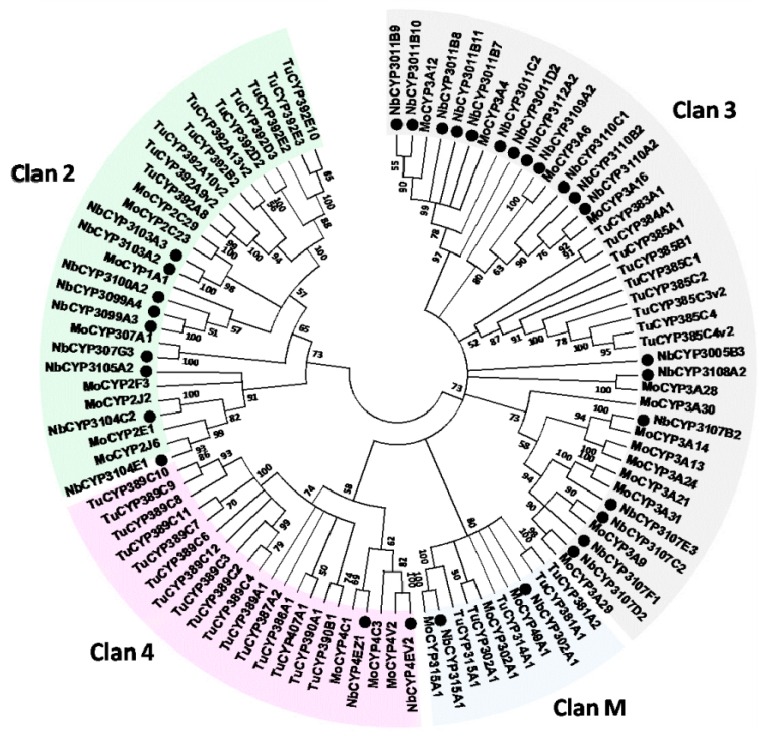
Neighbor-joining phylogenetic analysis of the *N. barkeri* P450 genes. Dots represent the *N. barkeri* P450 protein sequences. Nb: *N. barkeri*; Mo: *M. occidentalis*; Tu: *T. urticae*.

**Figure 3:**
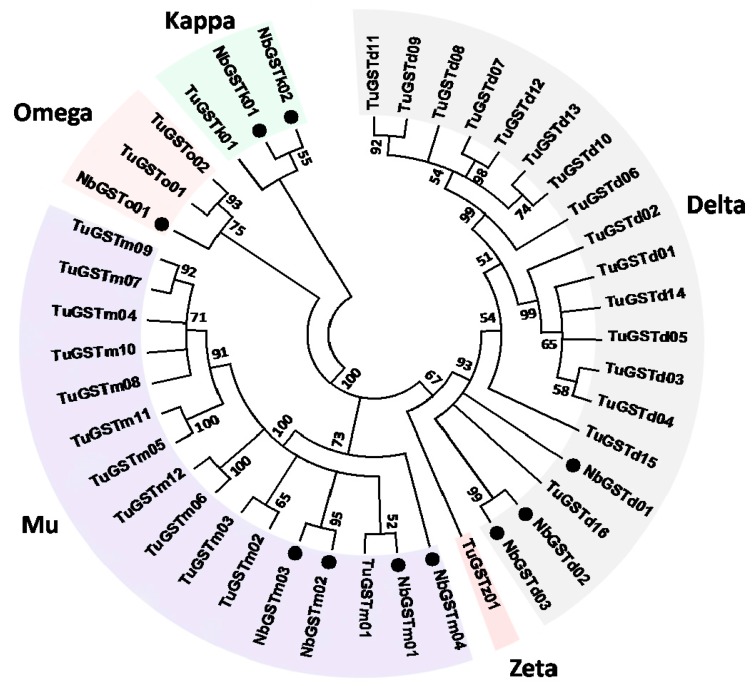
Neighbor-joining phylogenetic analysis of the *N. barkeri* GST genes. Dots represent the GST protein sequences from *N. barkeri*. Nb: *N. barkeri*; Tu: *T. urticae*.

In total, 43 P450 coding genes were identified from the nr database of this transcriptome. After removing short unigenes, 32 were used to construct the phylogenetic tree with other P450 genes generated from *Tetranychus urticae* (Figure 2) [26]. As demonstrated, 9, 19, 2, and 2 unigenes were classified into CYP 2, 3, and 4 Clan and mitochondrial Clan, respectively. Ten GST genes were also identified from the *N. barkeri* transcriptome. Among the GST sequences, 3, 2, 4 and 1 genes were classified into Delta, Kappa, Mu, and Omega class based on the phylogenetic analysis results with the GST genes from *T. urticae* (Figure 3).

**Figure 4:**
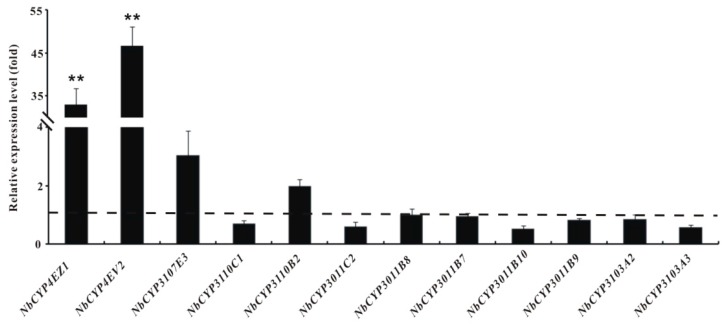
Fold-expression changes in selected P450s in the fenpropathrin resistant *N. barkeri* strain relative to its susceptible strain. Bars represent mean ± standard deviation. Asterisks indicate significant differences by *t*-test, ** *p* < 0.01.

**Figure 5:**
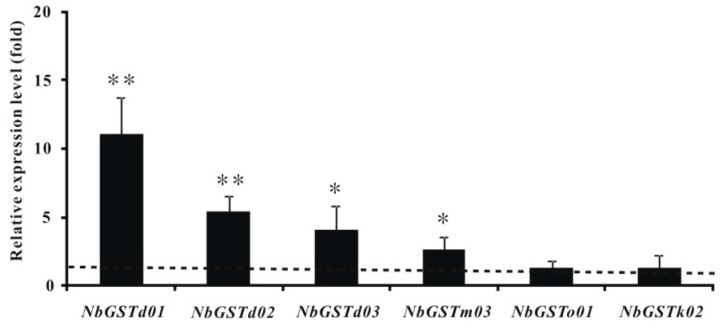
Fold-expression changes in selected GSTs in the fenpropathrinresistant *N. barkeri* strain relative to its susceptible strain. Bars represent mean ± standard deviation. Asterisks indicate significant difference by *t*-test, * *p* < 0.05 or ** *p* < 0.01.

To illuminate the expression patterns of those candidate genes, 12 P450 and 6 GST genes were selected randomly for quantitative PCR (*q*PCR) analysis. The expression levels of *NbCYP4EV2* and *NbCYP4EZ1* from CYP 4 Clan in the resistant strain were very significantly upregulated 46.69- and 32.90-fold (*p* < 0.01), respectively (Figure 4). The expression levels of *NbGSTd01*, *NbGSTd02*, and *NbGSTd03* from Delta class and *NbGSTm03* from Mu class of GSTs increased significantly 11.19-, 5.43-, 4.04-, and 3.64-fold, respectively (*p* < 0.05 and 0.01) (Figure 5).

## 3. Discussion

Biological control will undoubtedly become more important in integrated pest management. However, chemical insecticides are currently needed for pest control in the field. Thus, screening for chemical resistant natural enemies is a practical way to balance biological and chemical control. Nonetheless, the resistance mechanisms are poorly understood in those species, which is impeding development of this strategy. The main reason is the lack of genetic information. Therefore, we sequenced the transcriptome of a fenpropathrin resistant strain of *N. barkeri* and compared differences in gene sequences and expression patterns between fenpropathrin resistant and susceptible strains.

The fenpropathrin resistant *N. barkeri* strain was obtained, with a resistance ratio of 619.96-fold after several cycles (Table 1), indicating that this predatory mite has high fenpropathrin resistance under laboratory conditions, as reported in other predatory mites [12,13,14], and suggests that fenpropathrin resistance in *N. barkeri* may be a complex process, involving many genetic changes. 

The *N. barkeri* sodium channel was explored in detail in this study as the target site of pyrethroids after obtaining the ORF and verifying the point mutations. As expected, the putative *Nb*Sc protein sequence harbored typical conserved sodium channel domains, with six transmenbrane segments (S1–S6) in each of four hydrophobic regions (I–IV) (Figure S6) and shared very high similarity with homogeneous protein sequences from other species in the NCBI database, such as *M. occidentalis* (91.04%) and *V. destructo* (80.48%). However, the similarities were relatively low compared to that with *T. cinnabarinus* (52.13%) and *T. urticae* (44.89%). Moreover, the phylogenetic analysis further suggested that *Nb*Sc had a closer evolutionary relationship with *M. occidentalis* than with *V. destructo* and that the other sequences originated from Ixodiae and Tetranychidae (Figure 1).

Mutations in the sodium channel, so-called knockdown resistance (*kdr*), are essential for reducing sensitivity of the insect/mite nervous system to pyrethroids. Since the first *kdr* phenomenon was observed in houseflies in 1993 [27], more than 50 arthropods have been documented with one or more resistance-associated sodium mutations in the past two decades [28,29]. Although the mutated positions vary among species, most are found in transmembrane regions 4–6 of domain II [30,31] and in hydrophobic domain III [32,33]. Mutations in the linker regions between domain II and III are also found in pyrethroid-resistant *Plutella* *xylostella*, *T. urticae*, and *Anopheles gambiae* [28], which was also verified in our research (E1,233G and S1,282G). Co-occurring sodium channel mutations have a greater effect on reducing sensitivity to pyrethroids compared to a single mutation [34,35,36,37]. Co-occurring mutations nearly abolish sensitivity of the sodium channel or act as enhancers of existing mutations. In addition, synonymous mutations, nonsynonymous/synonymous mutation combinations, and the frequencies of mutations also contribute to different levels of resistance in insects [29,38,39]. However, only five Acarina mites, *V. destructor* [40], *T. evansi* [41], *T. urticae* [42], *T. cinnabarinus* [43], and *Panonychus citri* [44], have reported with sodium channel mutations, suggesting that further research on *kdr* resistance in mites is needed.

In addition to decreasing the sensitivity of the pyrethroid-target site, increasing the detoxification rate is the second major pyrethroid-resistance mechanism in arthropods. Thus, attention has been focused on P450 genes for their essential roles in xenobiotic metabolism and evolutionary adaptability. More than 3452 P450 genes have been submitted to NCBI [45,46,47]. However, little is known about *N. barkeri* P450s or those in other predatory mites. In this study, 43 P450 genes were identified from the transcriptome. The number of P450 genes in *N. barkeri* (*n* = 43) was quite low compared to those in *M. aeneus* (*n* = 77) [22], *L. entomophila* (*n* = 68) [19], *Bactrocera dorsalis* (*n* = 68) [48], *T. urticae* (*n* = 86) [26], and *P. citri* (*n* = 121) [49]. However, some of these P450 genes are believed to be vital in *N. barkeri* fenpropathrin metabolism. According to our results (Figure 4), *NbCYP4EV2* and *NbCYP4EZ1* are expected to be involved in chemical resistance due to their highly upregulated expression levels. This is also true in pyrethroid-resistant insects [50,51,52]. For example, the high expression levels of *CYP4M6*, *CYP4M7*, *CYP4L5*, and *CYP4L11* have been demonstrated to be the primary cause of deltamethrin-resistance in *Helicoverpa armigera* [53]. Moreover, *CYP325A3* in *A. gambiae* [54], *CYP4BN13V1* and *CYP4BN15* in *Leptinotarsa decemlineata* [55], and *CYP4G7* and *CYP345A1* in *Tribolium castaneum* [56] participate in permethrin, cyhalothrin, and cypermethrin resistance. Thus, it is reasonable to suspect that *NbCYP4EV2* and *NbCYP4EZ1* may play an important role in fenpropathrin metabolism in *N. barkeri*.

GSTs are also important detoxification enzymes associated with chemical resistance [57,58,59,60,61], which makes them a hotspot in entomology research. GSTs in insects are classified into Delta, Omega, Sigma, Zeta, Theta, and Epsilon class. Among these classes, Epsilon and Delta class are the largest and most unique classes in insects. However, the classification of GSTs in mites is quite different from that in insects. Similar to other mites [26,49,62], Mu class of *GST* genes (Figure 3), which is supposed to be exclusive to vertebrates, was identified in *N. barkeri*. However, no Sigma, Theta, Zeta, or Epsilon GST class genes were found in this dataset. In addition, Mu class was the largest GST gene group in *N. barkeri*, which differs from *T. urticae* [26]. Although the number and classification of GST genes vary among species, evidence has demonstrated that GSTs participate in chemical resistance. As mentioned before, increasing the detoxification rate by overexpression is a second major resistance mechanism in mites. Increased GST activities are observed at the protein level in abamectin [63,64] and spirodiclofen [65] resistant *T. urticae* and in fenpropathrin [66] and progargite resistant *T. cinnabarinus* [67]. Some Mu and Delta GST gene transcripts are highly expressed in chemical resistant *Sarcoptes scabiei* [68] and *T. cinnabarinus* [67]. The overexpressed GST genes from Mu, Epsilon, Theta, Delta, and Sigma class may participate in detoxification of beta-cypermethrin, deltamethrin, fenpropathrin, and lambda-cyhalothrin in *B. dorsalis* [69], *Locusta migratoria manilensis* [70], *P. xylostella* [71], *T. cinnabarinus* [62], and *Cydia pomonella* [72]. These findings suggest that *NbGSTd01*, *NbGSTd02,* and *NbGSTd03* from Delta class and *NbGSTm03* from Mu class may play essential roles in fenpropathrin metabolism in *N. barkeri* due to their highly elevated expression levels in the resistant strain (Figure 5).

Fenpropathrin resistance in *N. barkeri* may involve decreasing sensitivity of the fenpropathrin-targeted site and an increase in the detoxification rate at the same time, as described in *T. cinnabarinus* [43,62]. However, the trade-off is that chemical resistance allows arthropods a chance to survive but whether fecundity or life span is sacrificed is an interesting question for future study.

## 4. Materials and Methods

### 4.1. Colony Rearing and Maintenance

The original *N. barkeri* colony used in this study occurs naturally on lemon trees in an orchard of the Citrus Research Institute of Southwest University (Beibei, Chongqing, China; longitude 106°22′33′′ E/latitude 29°46′14′′ N). The colony was collected and has been maintained without pesticide exposure for dozens of generations in the laboratory on a sterilized artificial diet (wheat bran/flour = 9:1 weight ratio) infested with *Aleuroglyphus ovatus* at 25 ± 1 °C, 80% ± 5% relative humidity, and a 16:8 h (L:D) photoperiod. Based on preliminary bioassay results, this colony was used as the fenpropathrin susceptible strain due to its high sensitivity. 

### 4.2. Resistance Selection

Resistance selection was carried out simultaneously on prey (*A. ovatus*) and predators (*N. barkeri*). *A. ovatus* was fed a fenpropathrin-sprayed (150 mL/L artificial diet) artificial diet, as described previously [73]. After the flour mite population recovered, the selection was repeated with a higher dose. Routine resistance selection of *N. barkeri* was applied by transferring surviving predatory mites into a new rearing unit after treatment with a LC_50_ fenpropathrin solution for 24 h and supplied with fenpropathrin-resistant flour mites and the fenpropathrin-sprayed artificial diet. After the *N. barkeri* population increased to a certain level, the selection was repeated, and the LC_50_ dose was re-established as selection pressure.

### 4.3. Toxicity Testing and Determining the LC Value

The *N. barkeri* bioassay was conducted as described previously using simplified devices [14,74]. Forty coeval *N. barkeri* females were selected randomly and treated with a fenpropathrin solution for 5 s (200 g·L^−1^ EC; Sumitomo Chemical, Misawa, Japan), quickly dried with filter paper, and transferred to a Petri dish (9 cm in diameter) containing a 4 cm × 4 cm lemon leaf disc. The Petri dish was sealed with preservative film and a rubber band. The toxicity test included 7 serial fenpropathrin concentrations and one blank water treatment as a control, with three replications in each group. Lethal toxicity was evaluated after 24 h by counting the numbers of live and dead mites under a microscope fitted with an anatomical lens (SMZ-B4; Optec, Chongqing, China). Corrected mortality was calculated with Abbott’s formula to determine the LC_50_ value [75]. All trials were conducted under the conditions mentioned above.

### 4.4. RNA Preparation, Library Construction, and Sequencing

Different developmental stages of fenpropathrin-resistant *N. barkeri* (eggs, larva, nymphs, and adults) were collected separately, frozen in liquid nitrogen, and stored at −80 °C until use. Total RNA was extracted with the RNeasy^®^ Plus Micro Kit (Qiagen, Hilden, Germany). RNA quality and integrity were determined with the NanoDrop 2000 UV-Vis Spectrophotometer (ThermoScientific, Wilmington, DE, USA) and gel electrophoresis. The Truseq^®^ RNA Sample Preparation Kit (Illumina, San Diego, CA, USA) was employed to prepare the library, which was sequenced on an Hiseq™2000 (Illumina) platform using the paired-end method. All manipulations followed the manufacturer’s specifications.

### 4.5. Sequence Analysis, de Novo Assembly, and Identification of Interesting Genes

After filtering the low quality reads, the qualified short reads were assembled and gap-filled into contigs, transcripts, and unigenes using the Trinity *de novo* program step-by-step [76]. Then, the assembled unigenes were screened against the NCBI nr, Swiss-Prot, KEGG, COG, and GO databases with a threshold *E*-value <10^−5^ to predict sequence direction, metabolic pathway, and potential function. The *N. barkeri* transcriptome dataset was submitted to the NCBI Short Read Archive (SRA), with accession number: SRR2924862.

The sodium channel, P450, and GST sequences were regarded as interesting genes and searched against the nr dataset with a cut-off *E*-value <10^−5^. Unigenes with the same BLAST results or with high similarity to each other were considered the same gene. The results were double-checked using ORF finder [77], and BLASTP. MEGA 5.04 was used to analyze the phylogenetic relationships between these interesting genes and related sequences in other species, such as *T. urticae*, *M. occidentalis*, and *V. destructor*, to predict their classification using the neighbor-joining method with 1000 bootstrap replications.

### 4.6. Cloning and Mutations Confirmation of Sodium Channel Gene

To obtain the full coding region of the *NbSc*, reverse transcription PCR (RT-PCR) was carried out with eight pairs of gene-specific primers (*NbSc*-A-F/R—*NbSc*-H-F/R, Table S1, Supplementary Material), which were designed based on three VGSC-related unigenes (comp28931_c2, comp28931_c3, and comp28696_c0) screened from the transcriptome. Total RNA was extracted from the *N. barkeri* fenpropathrin-resistant and susceptible strains, as mentioned above. Then, 1 μg total RNA was used to synthesize first-strand cDNA with the PrimeScript^™^ RT reagent Kit (TaKaRa Bio, Dalian, China). The 25 μL PCR reaction system included: 1.0 μL cDNA template, 1.0 μL of each primer (10 μM), 2.0 μL dNTPs (2.5 mM each), 2.5 μL Mg^2+^ (25 mM), 10× PCR buffer (Mg^2+^ free), 15.0 μL nuclease-free water, and 0.25 μL of *Pyrobest* DNA Polymerase (5 U/μL) (TaKaRa Bio). All PCR reactions were conducted under the following conditions: 3 min at 94 °C, 35 cycles of 30 s at 94 °C, 30 s at 53–62 °C, 1 min at 72 °C, and final extension at 72 °C for 5 min. The target PCR products were purified and cloned into the pMD-19T vector (TaKaRa Bio) and sequenced (Invitrogen, Shanghai, China). The DNAMAN 5.2.2 (Lynnon, Quebec, QC, Canada) and ClustalW2 [78] programs were adopted to assemble the full-length cDNA sequence and predict its molecular features and conserved domains. A single-nucleotide polymorphism analysis was carried out to detect possible point mutations in *NbSc* with specific primers (*NbSc*-M-F/R, Table S1). Clustal X was applied to analyze similarity between the *Nb*Sc sequences generated from the fenpropathrin resistant and susceptible strains.

### 4.7. Differential Expression Analysis of P450s and GSTs

*q*PCR was used to evaluate the P450 and GST gene expression profiles in the *N. barkeri* fenpropathrin resistant and susceptible strains. Primers for each selected gene (Table S2, Supplementary Material) were designed with Primer 3 [79] and generated 83–145 bp long fragments. The 20 μL reaction mixture contained 10 μL SYBR *Premix* Ex Taq™ II (TaKaRa Bio), 1 μL of each primer (10 μM), 1 μL cDNA, and 7 μL nuclease-free water. The *q*PCR assay was validated on the iCycler iQ™ Multi-Color Real-Time PCR Detection System (Bio-Rad, Hercules, CA, USA) with the following thermal cycling conditions: 95 °C for 2 min, followed by 40 cycles of 95 °C for 30 s, 60 °C for 30 s, and 60–95 °C for the melting curve. Mean relative expression levels of the target genes were calculated using three independent biological samples from the fenpropathrin-resistant and susceptible strains using the 2^−ΔΔ*C*t^ method [80] and the β*-actin* gene (GenBank accession number: KP310115, *NbBactin*-Q-F/R, Table S2) validated as the optimal reference gene. The expression level of all interesting genes in the susceptible strain was arbitrarily treated as 1. Analysis of variance and the *t*-test were performed to compare the data with the SAS 8.01 program (SAS Institute, Cary, NC, USA). A *p*-value < 0.05 was considered significant. Results are reported as mean ± standard deviation.

## 5. Conclusions

The results demonstrate that *N. barkeri* achieved high pyrethroid resistance by increasing detoxification rates (P450s and GSTs) and decreasing sensitivity of the target site (sodium channel) simultaneously. Our results suggest that fenpropathrin resistance is a complex biological process involving many genetic changes and provide new insight into the resistance mechanism in *N. barkeri*.

## Figures and Tables

**Table 1:** Resistance selection of *N. barkeri* against fenpropathrin.

Strains	Regression Equation	*X*^2^	95% Confidence Interval (mg/L)	*R*^2^	Resistance Ratio
F_S_	*Y* = 3.2619 + 1.1186*X*	2.0824	35.79 (28.64–44.73)	0.996	1
F_R_	*Y* = 0.9489 + 0.9321*X*	0.0574	22,190.91 (2853.80–172,554.97)	0.998	619.96

F_S_: fenpropathrin susceptible strain; F_R_: fenpropathrin resistant strain.

**Table 2:** Primary description of the *N. barkeri* transcriptome.

Sequencing	
Total number of reads	25,192,607
Total number of nucleotides (nt)	5,087,856,659
GC percentage (%)	50.85
Q20 percentage (%)	100.00
N percentage (%)	0.01
Number of contigs	1,385,792
Mean length of contigs (bp)	74.76
Number of transcripts	50,462
Mean length of transcripts (bp)	1494.58
N50 of transcripts (bp)	3007
Number of unigenes	34,211
Mean length of unigenes (bp)	1016.52
N50 of unigenes (bp)	2126
Total annotated unigenes	15,987
Unigenes annotations against nr	15,866
Unigenes annotations against Swiss-Prot	10,486
Unigenes annotations against KEGG	5445
Unigenes annotations against COG	5673
Unigenes annotations against GO	8707

GC percentage: proportion of G and C nucleotides of all nucleotides; Q20 percentage: proportion of nucleotides in which the quality value was >20; N percentage: percentage of unknown nucleotides in clean reads; N50: transcript length measurement (or unigene length), indicating that 50% of all assembly residues in the transcripts (or unigenes) had a length of at least X.

## Supplementary Materials

Supplementary materials can be found at http://www.mdpi.com/1422-0067/17/6/704/s1.

## Abbreviations

COG	Cluster of Orthologous Groups
GO	Gene Ontology
GST	Glutathione S-transferase
KEGG	Kyoto Encyclopedia of Genes and Genomes
LC_50_	Lethal concentration 50
ORF	Open reading frame
nr	Nonredundant
P450	Cytochrome P450
*q*PCR	Quantitative PCR
RT-PCR	Reverse transcription PCR
VGSC	Voltage-gated sodium channel

## References

[1] Wu W.N., Ou J.F., Huang J.L., Huo C.Y., Wu L.L. (2009). Arachnida: Acari: Phytoseiidae. Fauna Sinica: Invertebrata.

[2] Fan Y.Q., Petitt F.L. (1994). Functional response of *Neoseiulus barkeri* Hughes on two-spotted spider mite (Acari: Tetranychidae). Exp. Appl. Acarol..

[3] Fan Y.Q., Petitt F.L. (1994). Biological control of broad mite, *Polyphagotarsonemus latus* (Banks), by *Neoseiulus barkeri* Hughes on pepper. Biol. Control..

[4] Fernando L.C.P., Aratchige N.S., Kumari S.L.M.L., Appuhamy P.A.L.D., Hapuarachchi D.C.L. (2004). Development of a method for mass rearing of *Neoseiulus baraki*, a mite predatory on the coconut mite, *Aceria guerreronis*. Cocos.

[5] Bakker F.M., Sabelis M.W. (1989). How larvae of *Thrips tabaci* reduce the attack success of phytoseiid predators. Entomol. Exp. Appl..

[6] Hansen L.S. (1988). Control of *Thrips tabaci* (*Thysanoptera:Thripidae*) on glasshouse cucumber using large introductions of predatory mites *Amblyseius barkeri* (*Acarina:Phytoseiidae*). Biocontrol.

[7] Hessein N.A., Parrella M.P. (1990). Predatory mites help control thrips on floriculture crops. Calif. Agric..

[8] BrØdsgaard H.F., Hansen L.S. (1992). Effect of *Amblyseius cucumeris* and *Amblyseius barkeri* as biological control agents of *Thrips tabaci* on glasshouse cucumbers. Biocontrol. Sci. Technol..

[9] Peña J.E., Obsorne L. (1996). Biological control of *Polyphagotarsonemus latus* (Acarina: Tarsonemidae) in greenhouses and field trials using introductions of predacious mites (Acarina: Phytoseiidae). Entomophaga.

[10] Nomikou M., Janssen A., Schraag R., Sabelis M.W. (2001). Phytoseiid predators as potential biological control agents for *Bemisia tabacci*. Exp. Appl. Acarol..

[11] Ling P., Xia B., Li P.X., Shu C., Zhong L., Li A.H. (2008). Functional response of *Amblyseius barkeri* (Acarina: Phytoseiidae) on *Panonychus citri* (Acari: Tetranychidae). Acta Arachnol. Sin..

[12] Sato M.E., Miyata T., Kawai A., Nakano O. (2000). Selection for resistance and susceptibility to methidathion and cross resistance in *Amblyseius. wormersleyi* Schicha (Acari: Phytoseiidae). Appl. Entomol. Zool..

[13] Auger P., Bonafos R., Kreiter S., Delorme R. (2005). A genetic analysis of mancozeb resistance in *Typhlodromus pyri* (Acari: Phytoseiidae). Exp. Appl. Acarol..

[14] Salman S.Y., Recep A.Y. (2013). Analysis of hexythiazox resistance mechanisms in a laboratory selected predatory mite *Neoseiulus californicus* (Acari: Phytoseiidae). Turk. Entomol. Derg. Tu..

[15] Hoy M.A. (1982). Large-scale releases of pesticide-resistant spider mite predators. Calif. Agric..

[16] Hoy M.A., Barnett W.W., Hendricks L.C., Castro D., Cahn D., Bentley W.J. (1984). Managing spider mites in almonds with pesticide-resistant predators. Calif. Agric..

[17] Hluchý M., Zacharda M., Gradt P. (1996). Results of large-scale releases of predatory phytoseiid mite, *Typhlodromus pyri*, an OP-resistance population Mikulov, in apple or chards in South Tyrol and France. Acta Hortic..

[18] Casida J.E., Durkin K.A. (2013). Neuroactive insecticides: Targets, selectivity, resistance, and secondary effects. Annu. Rev. Entomol..

[19] Wei D.D., Chen E.H., Ding T.B., Chen S.C., Dou W., Wang J.J. (2013). *De novo* assembly, gene annotation, and marker discovery in stored-product pest *Liposcelis entomophila* (Enderlein) using transcriptome sequences. PLoS ONE.

[20] Zhang Z.J., Zhang P.J., Li W.D., Zhang J.M., Huang F., Yang J., Bei Y., Lu Y. (2013). *De novo* transcriptome sequencing in *Frankliniella occidentalis* to identify genes involved in plant virus transmission and insecticide resistance. Genomics.

[21] Chen E.H., Wei D.D., Shen G.M., Yuan G.R., Bai P.P., Wang J.J. (2014). *De novo* characterization of the *Dialeurodes citri* transcriptome: Mining genes involved in stress resistance and simple sequence repeats (SSRs) discovery. Insect Mol. Biol..

[22] Zimmer C.T., Maiwald F., Schorn C., Bass C., Ott M.C., Nauen R. (2014). A *de novo* trancriptome of European pollen beetle populations and its analysis, with special reference to insecticide action and resistance. Insect Mol. Biol..

[23] Liu Y., Wu H.Y., Xie Q., Bu W.J. (2015). Novel detection of insecticide resistance related P450 genes and transcriptome analysis of the hemimetabolous pest *Erthesina fullo* (Thunberg) (Hemiptera: Heteroptera). PLoS ONE.

[24] Hoy M.A., Yu F.H., Meyer J.M., Tarazona O.A., Jeyaprakash A., Wu K. (2013). Transcriptome sequencing and annotation of the predatory mite *Metaseiulus occidentalis* (Acari: Phytoseiidae): A cautionary tale about possible contamination by prey sequences. Exp. Appl. Acarol..

[25] Cabrera A.R., Donohue K.V., Khalil S.M.S., Scholl E., Opperman C., Sonenshine D.E., Roe R.M. (2011). New approach for the study of mite reproduction: The first transcriptome analysis of a mite, *Phytoseiulus. persimilis* (Acari: Phytoseiidae). J. Insect Physiol..

[26] Grbić M., van Leeuween T., Clark R.M., Rombauts S., Rouzé P., Grbić V., Osborne E.J., Dermauw W., Thi Ngoc P.C., Ortego F. (2011). The genome of *Tetranychus urticae* reveals herbivorous pest adaptations. Nature.

[27] Williamson M.S., Denholm I., Bell C.A., Devonshire A.L. (1993). Knockdown resistance (*kdr*) to DDT and pyrethroid insecticides maps to a sodium channel gene locus in the housefly (*Musca domestica*). Mol. Gen. Genet..

[28] Rinkevich F.D., Du Y.Z., Dong K. (2013). Diversity and convergence of sodium channel mutations involved in resistance to pyrethroids. Pestic. Biochem. Physiol..

[29] Dong K., Du Y.Z., Rinkevich F., Nomura Y., Xu P., Wang L.X., Kristopher S., Boris S.Z. (2014). Molecular biology of insect sodium channels and pyrethroid resistance. Insect Biochem. Mol. Biol..

[30] Soderlund D.M., Knipple D.C. (2003). The molecular biology of knockdown resistance to pyrethroid insecticides. Insect Biochem. Mol. Biol..

[31] Davies T.E., O’Reilly A.O., Field L.M., Wallace B.A., Williamson M.S. (2008). Knockdown resistance to DDT and pyrethroids: From target-site mutations to molecular modelling. Pest Manag. Sci..

[32] Wang R., Liu Z., Dong K., Elzen P.J., Pettis J., Huang Z.Y. (2002). Association of novel mutations in a sodium channel gene with fluvalinate resistance in the mite, *Varroa destructor*. J. Apic. Res..

[33] Tsagkarakou A., Van Leeuwen T., Khajehali J., Ilian A., Grispou M., Williamson M.S., Tirry L., Vontas J. (2009). Identification of pyrethroid resistance associated mutations in the para sodium channel of the two-spotted spider mite *Tetranychus urticae* (Acari: Tetranychidae). Insect Mol. Biol..

[34] Vais H., Williamson M.S., Goodson S.J., Devonshire A.L., Warmke J.W., Usherwood P.N., Cohen C.J. (2000). Activation of *Drosophila* sodium channels promotes modification by deltamethrin-reductions in affinity caused by knock-down resistance mutations. J. Gen. Physiol..

[35] Lee S.H., Smith T.J., Knipple D.C., Soderlund D.M. (1999). Mutations in the house fly *Vssc1* sodium channel gene associated with *super-kdr* resistance abolish the pyrethroid sensitivity of Vssc1/tipE sodium channels expressed in *Xenopus* oocytes. Insect Biochem. Mol. Biol..

[36] Kyong S.Y., Symington S.B., Si H.L., Soderlund D.M., Clark J.M. (2008). Three mutations identified in the voltage-sensitive sodium channel alpha-subunit gene of permethrin-resistant human head lice reduce the permethrin sensitivity of house fly Vssc1 sodium channels expressed in *Xenopus.* oocytes. Insect Biochem. Mol. Biol..

[37] Tan J., Liu Z., Tsai T.D., Valles S.M., Goldin A.L., Dong K. (2002). Novel sodium channel gene mutations in *Blattella. germanica* reduce the sensitivity of expressed channels to deltamethrin. Insect Biochem. Mol. Biol..

[38] Xu Q., Zhang L., Li T., Zhang L., He L., Dong K., Liu N.N. (2012). Evolutionary adaptation of the amino acid and codon usage of the mosquito sodium channel following insecticide selection in the field mosquitoes. PLoS ONE.

[39] Alvarez L.C., Ponce G., Saavedra-Rodriguez K., Lopez B., Flores A.E. (2015). Frequency of V1016I and F1534C mutations in the voltage-gatedsodiumchannelgene in *Aedes aegypti* in Venezuela. Pest Manag. Sci..

[40] González-Cabrera J., Davies T.G., Field L.M., Kennedy P.J., Williamson M.S. (2013). An amino acid substitution (L925V) associated with resistance to pyrethroids in *Varroa destructor*. PLoS ONE.

[41] Nyoni B.N., Gorman K., Mzilahowa T., Williamson M.S., Navajas M., Field L.M., Bass C. (2011). Pyrethroid resistance in the tomato red spider mite, *Tetranychus evansi*, is associated with mutation of the *para*-type sodium channel. Pest Manag. Sci..

[42] Kwon D.H., Clark J.M., Lee S.H. (2010). Cloning of a sodium channel gene and identification of mutations putatively associated with fenpropathrin resistance in *Tetranychus urtica*. Pestic. Biochem. Physiol..

[43] Feng Y.N., Zhao S., Sun W., Li M., Lu W.C., He L. (2011). The sodium channel gene in *Tetranychus cinnabarinus* (Boisduval): Identification and expression analysis of a mutation associated with pyrethroid resistance. Pest Manag. Sci..

[44] Ding T.B., Zhong R., Jiang X.Z., Liao C.Y., Xia W.K., Liu B., Dou W., Wang J.J. (2015). Molecular characterisation of asodiumchannelgene and identification of a Phe1538 to Ile mutation in citrus red mite, *Panonychus citri*. Pest Manag. Sci..

[45] Ai J.W., Zhu Y., Duan J., Yu Q.Y., Zhang G.J., Wan F., Xiang Z.H. (2011). Genome-wide analysis of cytochrome P450 monooxygenase genes in the silkworm, *Bombyx mori*. Gene.

[46] Feyereisen R. (2011). Arthropod CY Pomes illustrate the tempo and mode in P450 evolution. Biochim. Biophys. Acta.

[47] Yang T., Liu N.N. (2011). Genome analysis of cytochrome P450s and their expression profiles in insecticide resistant mosquitoes, *Culex quinquefasciatus*. PLoS ONE.

[48] Shen G.M., Dou W., Niu J.Z., Jiang H.B., Yang W.J., Jia F.X., Hu F., Cong L., Wang J.J. (2011). Transcriptome analysis of the oriental fruit fly *Bactrocera dorsalis*. PLoS ONE.

[49] Liu B., Jiang G.F., Zhang Y.F., Li J.L., Li X.J., Yue J.S., Chen F., Liu H.Q., Li H.J., Ran C. (2011). Analysis of transcriptome differences between resistant and susceptible strains of the citrus red mite *Panonychus citri* (Acari: Tetranychidae). PLoS ONE.

[50] Avicor S.W., Wajidi M.F.F., El-Garj F.M.A., Jaal Z., Yahaya Z.S. (2014). Insecticidal activity and expression of cytochrome P450 family 4 genes in *Aedes. albopictus* after exposure to pyrethroid mosquito coils. Protein J..

[51] Killiny N., Hajeri S., Tiwari S., Gowda S., Stelinski L.L. (2014). Double-stranded RNA uptake through topical application, mediates silencing of five *CYP4* genes and suppresses insecticide resistance in *Diaphorina citri*. PLoS ONE.

[52] Rasool A., Joußen N., Lorenz S., Ellinger R., Schneider B., Khan S.A., Ashfaq M., Hechel D.G. (2014). An independent occurrence of the chimeric P450 enzyme CYP337B3 of *Helicoverpa armigera* confers cypermethrin resistance in Pakistan. Insect Biochem. Mol. Biol..

[53] Brun-Barale A., Hèma O., Martin T., Suraporn S., Audant P., Sezutsu H., Feyereisen R. (2010). Multiple P450 genes overexpressed in deltamethrin-resistant strains of *Helicoverpa armigera*. Pest Manag. Sci..

[54] David J.P., Strode C., Vontas J., Nikou D., Vaughan A., Pignatelli P.M., Louis C., Hemingway J., Ranson H. (2005). The *Anopheles gambiae* detoxification chip: A highly specific microarray to study metabolic-based insecticide resistance in malaria vectors. Proc. Natl. Acad. Sci. USA.

[55] Wan P.J., Shi X.Q., Kong Y., Zhou L.T., Guo W.C., Ahmat T., Li G.Q. (2013). Identification of cytochrome P450 monooxygenase genes and their expression profiles in cyhalothrin-treated Colorado potato beetle, *Leptinotarsa decemlineata*. Pestic. Biochem. Physiol..

[56] Liang X., Xiao D., He Y.P., Yao J.X., Zhu G.N., Zhu K.Y. (2015). Insecticide-mediated up-regulation of cytochrome P450 genes in the red flour beetle (*Tribolium castaneum*). Int. J. Mol. Sci..

[57] Sonoda S., Tsumuki H. (2005). Studies on glutathione S-transferase gene involved in chlorfluazuron resistance of the diamondback moth, *Plutella xylostella* L. (Lepidoptera: Yponomeutidae). Pestic. Biochem. Physiol..

[58] Ding Y., Ortelli F., Rossiter L.C., Hemingway J., Ranson H. (2003). The *Anopheles gambiae* glutathione transferase supergene family: Annotation, phylogeny and expression profiles. BMC Genom..

[59] Samra A.I., Kamita S.G., Yao H.W., Cornel A.J., Hammock B.D. (2012). Cloning and characterization of two glutathione S-transferases from pyrethroid resistant *Culex pipiens*. Pest Manag. Sci..

[60] Tang B.Z., Sun J.Y., Zhou X.G., Gao X.W., Liang P. (2011). The stability and biochemical basis of fufenozide resistance in a laboratory-selected strain of *Plutella xylostella*. Pestic. Biochem. Physiol..

[61] You Y.C., Xie M., Ren N.N., Cheng X.M., Li J.Y., Ma X.L., Zou M.M., Liette V., Geoff M.G., You M.S. (2015). Characterization and expression profiling of glutathione S-transferases in the diamondback moth, *Plutella xylostella* (L.). BMC Genom..

[62] Shen G.M., Shi L., Xu Z.F., He L. (2014). Inducible expression of Mu-class glutathione S-transferases is associated with fenpropathrin resistance in *Tetranychus cinnabarinus*. Int. J. Mol. Sci..

[63] Nauen R., Stumpf N. (2002). Fluorometric microplate assay to measure glutathione S-transferase activity in insects and mites using monochlorobimane. Anal. Biochem..

[64] Pavlidi N., Tseliou V., Riga M., Nauen R., Van Leeuwen T., Labrou N.E., Vontas J. (2015). Functional characterization ofglutathioneS-transferases associated with insecticide resistance in *Tetranychus urticae*. Pestic. Boichem. Physiol..

[65] Rauch N., Nauen R. (2002). Spirodiclofen resistance risk assessment in *Tetranychus urticae* (Acari: Tetranychidae): A biochemical approach. Pestic. Biochem. Physiol..

[66] He L., Xue C.H., Wang J.J., Li M., Lu W.C., Zhao Z.M. (2009). Resistance selection and biochemical mechanism of resistance to two Acaricides in *Tetranychus cinnabarinus* (Boiduval). Pestic. Biochem. Physiol..

[67] Luo Y.J., Yang Z.G., Xie D.Y., Ding W., Da A.S., Ni J., Chai J.P., Huang P., Jiang X.J., Li S.X. (2014). Molecular cloning and expression of glutathione S-transferases involved in propargite resistance of the carmine spider mite, *Tetranychus cinnabarinus* (Boisduval). Pestic. Biochem. Physiol..

[68] Mounsey K.E., Pasay C.J., Arlian L.G., Morgan M.S., Holt D.C., Currie B.J., Currie B.J., Walton S.F., McCarthy J.S. (2010). Increased transcription of glutathione S-transferases in acaricide exposed scabies mites. Parasites Vectors.

[69] Hu F., Dou W., Wang J.J., Jia F.X., Wang J.J. (2013). Multiple glutathione S-transferase genes: Identification and expression in oriental fruit fly, *Bactrocera dorsalis*. Pest Manag. Sci..

[70] Qin G.H., Jia M., Liu T., Xuan T., Zhu K.Y., Guo Y.P., Ma E.B., Zhang J.Z. (2011). Identification and characterisation of ten glutathione S-transferase genes from oriental migratory locust, *Locusta migratoria manilensis* (Meyen). Pest Manag. Sci..

[71] Chen X.E., Zhang Y.L. (2015). Identification and characterisation of multiple glutathione S-transferase genes from the diamondback moth, *Plutella xylostella*. Pest Manag. Sci..

[72] Liu J.Y., Yang X.Q., Zhang Y.L. (2014). Characterization of a lambda-cyhalothrin metabolizing glutathione S-transferase *CpGSTd1* from *Cydia pomonella* (L.). Appl. Microbiol. Biotechnol..

[73] Petrushov A.Z. (1992). Pyrethroid resistance in the predacious mite *Amblyseius barkeri*. EPPO Bull..

[74] Duso C., Malagnini V., Pozzebon A., Buzzetti F.M., Tirello P. (2008). A method to assess the effects of pesticides on the predatory mite *Phytoseiulus persimilis* (Acari Phytoseiidae) in the laboratory. Biocontrol. Sci. Technol..

[75] Abbott W.S. (1925). A method of computing the effectiveness of an insecticide. J. Am. Mosq. Control Assoc..

[76] Grabherr M.G., Haas B.J., Yassour M., Levin J.Z., Thompson D.A., Amit I., Fan L., Raychowdhury R., Zeng Q.D., Chen Z.H. (2011). Full-length transcriptome assembly from RNA-Seq data without a reference genome. Nat. Biotechnol..

[77] ORF finder. http://www.ncbi.nlm.nih.gov/gorf/gorf.html.

[78] ClustalW2. http://www.ebi.ac.uk/Tools/msa/clustalw2/.

[79] Primer 3. http://primer3.ut.ee/.

[80] Livak K.J., Schmittgen T.D. (2001). Analysis of relative gene expression data using real-time quantitative PCR and the 2^−ΔΔ*C*t^ method. Methods.

