# Supplementary material for: Transcriptome and Difference Analysis of Fenpropathrin Resistant Predatory Mite, Neoseiulus barkeri (Hughes)
Source: Int J Mol Sci. 2016 May 27;17(6):704. doi: 10.3390/ijms17060704 (PMC4926325; doi:10.3390/ijms17060704)
Supplement: Supplementary file 1 [file ijms-17-00704-s001.pdf]

# Supplementary Materials: Transcriptome and Difference Analysis of Fenpropathrin Resistant Predatory Mite, *Neoseiulus barkeri* (Hughes)

**Lin Cong, Fei Chen, Shijiang Yu, Lili Ding, Juan Yang, Ren Luo, Huixia Tian, Hongjun Li, Haoqiang Liu and Chun Ran**

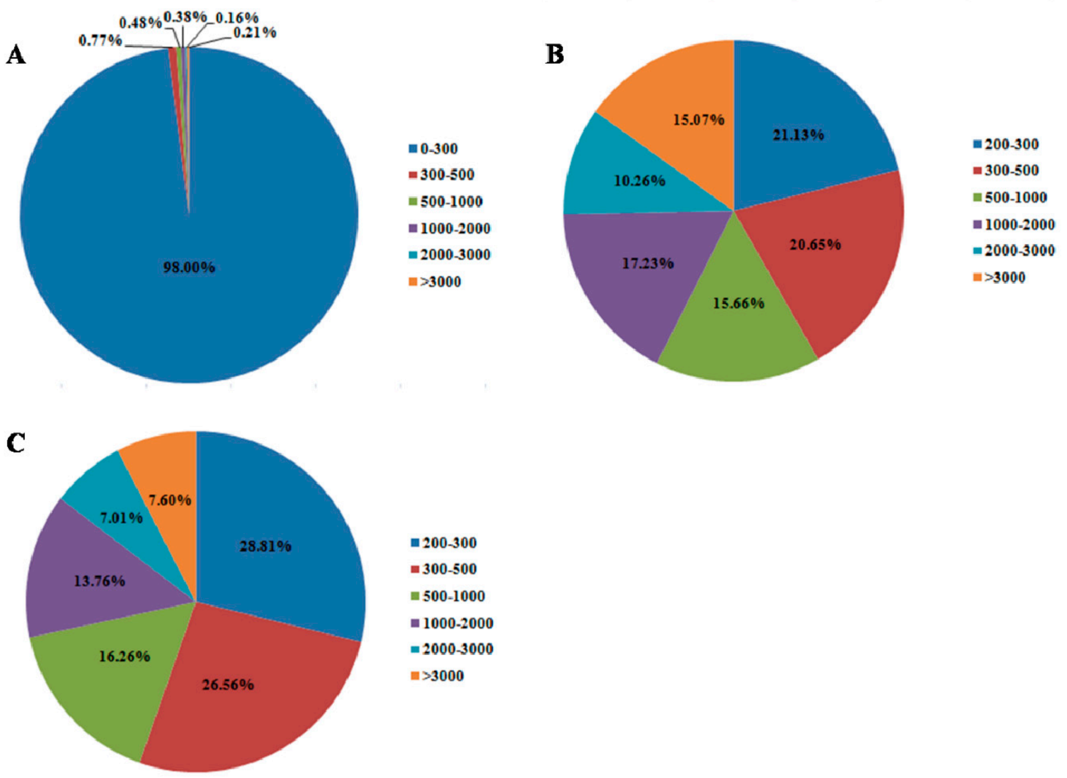


**Figure S1.** Length distributions of *N. barkeri* transcriptome data. (**A**) Length distribution of contig sequences; (**B**) length distribution of transcript sequences; (**C**) length distribution of unigene sequences.

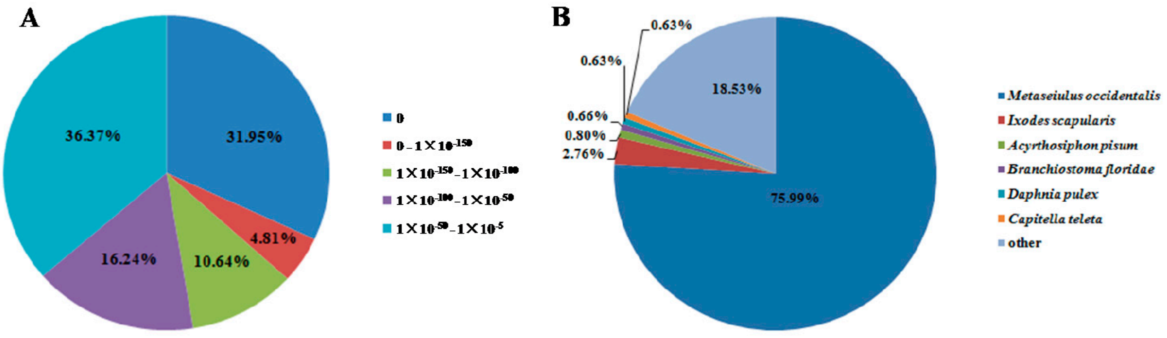


**Figure S2.** *E*-values and distributions of the *N. barkeri* unigenes. (**A**) E-value distribution of BLASTX hits in the non-redundant (nr) database for unigenes (cut-off $E < 10^{-5}$); (**B**) species distribution.

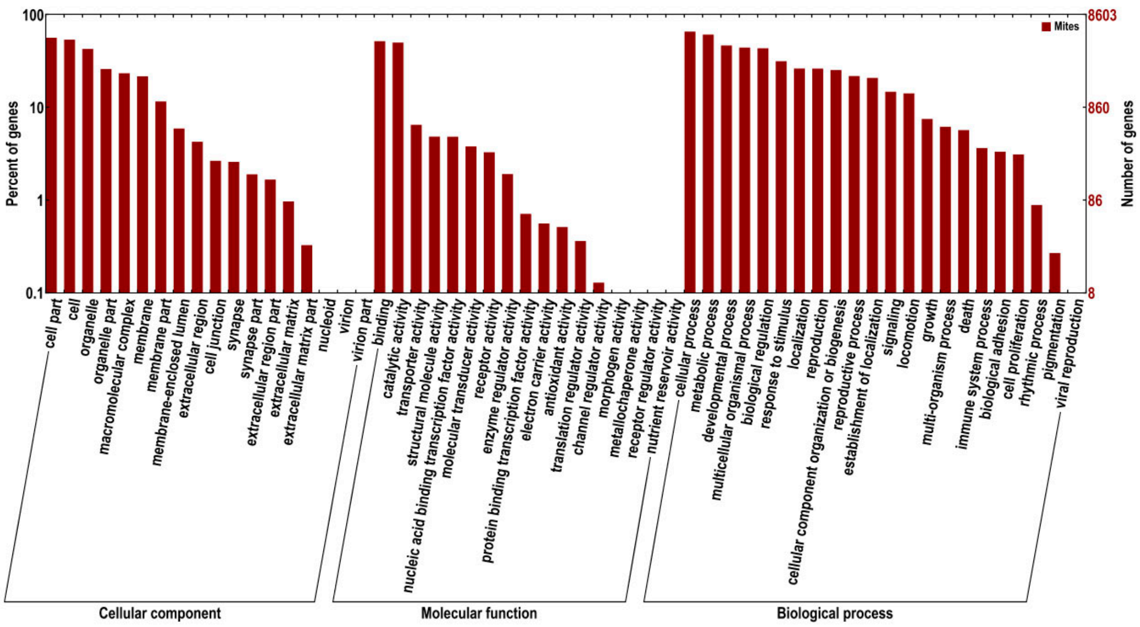


**Figure S3.** Gene Ontology (GO) analysis of the *N. barkeri* transcriptome.

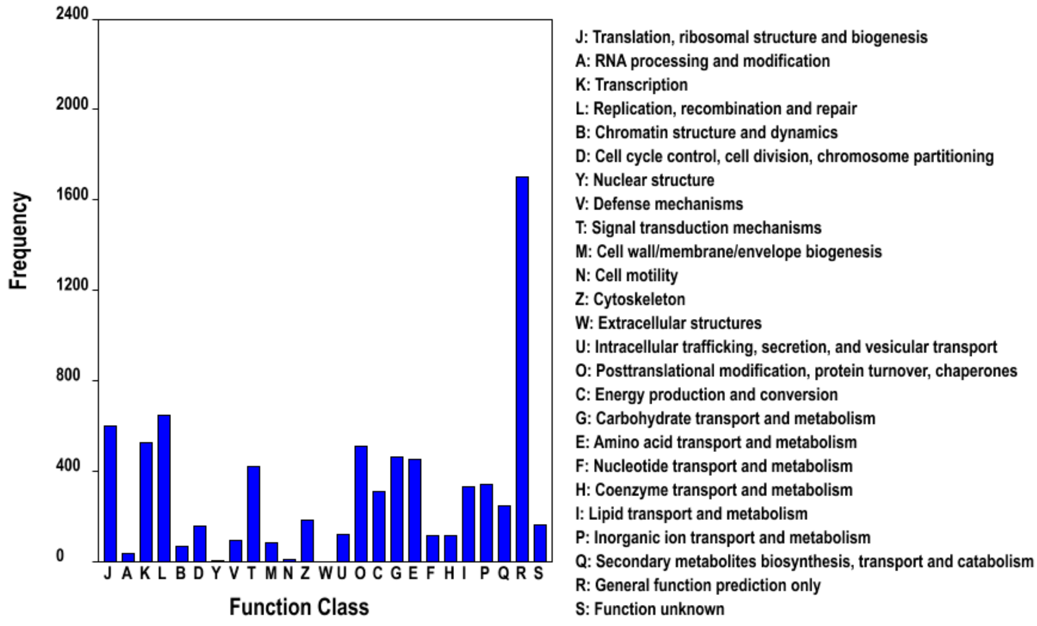


**Figure S4.** Classification of the Cluster of Orthologous Groups (COG) analysis of the *N. barkeri* transcriptome.

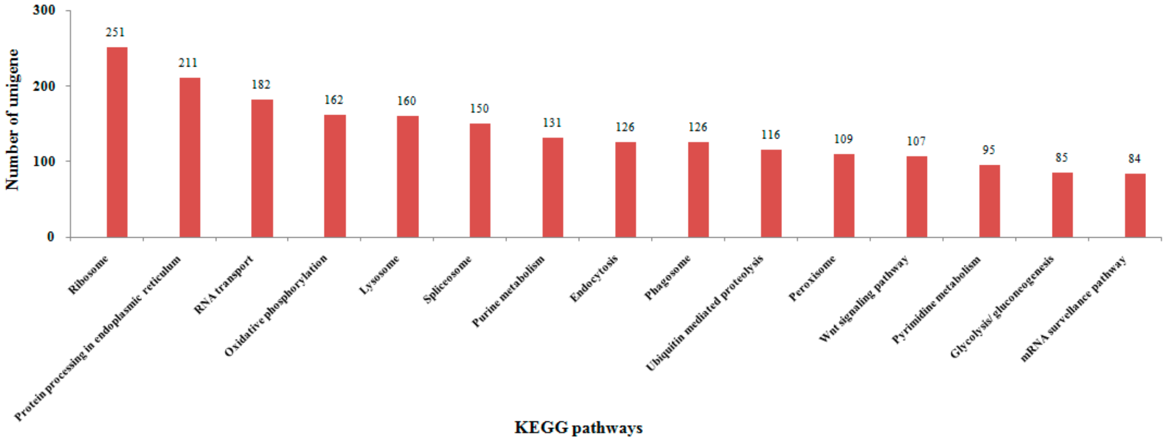


**Figure S5.** Kyoto Encyclopedia of Genes and Genomes (KEGG) annotation of the *N. barkeri* transcriptome. The top 15 pathways are shown.

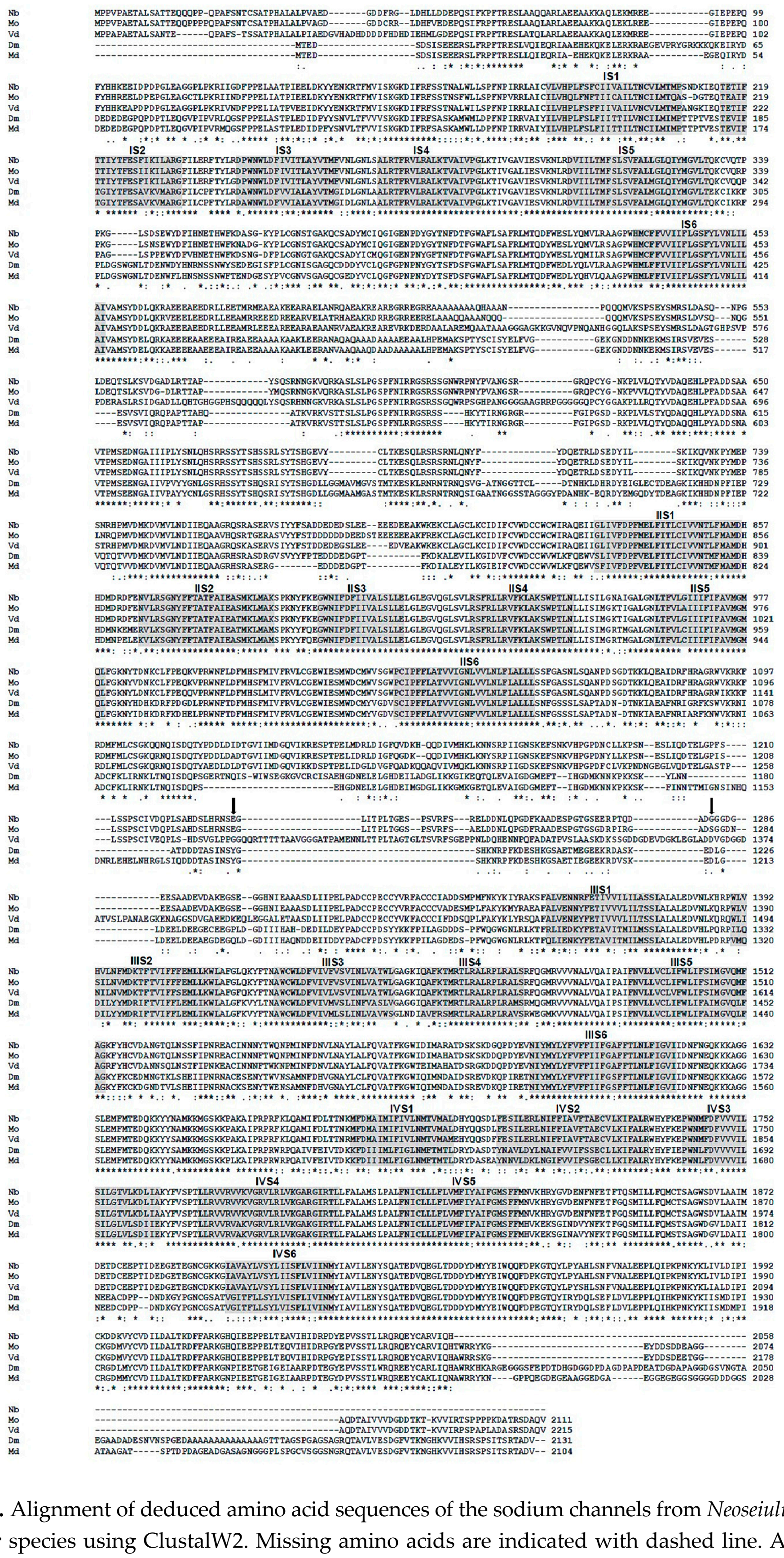

**Figure S6.** Alignment of deduced amino acid sequences of the sodium channels from *Neoseiulus barkeri* and other species using ClustalW2. Missing amino acids are indicated with dashed line. Asterisks, colons, and periods represent fully, strongly, and weakly conserved residues, respectively. The transmembrane regions (S1–S6) in the hydrophobic domains (I–IV) are shadowed. The locations of the mutations are indicated with "↓". Nb: *N. barkeri*; Mo: *M. occidentalis*; Vd: *V. destructor*; Dm: *D. melanogaster*; Md: *M. domestica*.

**Table S1.** Primers used to isolate the full-length *N. barkeri* sodium channel (*NbSc*) cDNA sequence and verify the *N. barkeri* mutations.

| Primers | Primer Sequences (5′-3′) | Length of Fragments (bp) |
|---|---|---|
| *NbSc*-A-F | GCCGAGACTGCTTTGTCAGC | 823 |
| *NbSc*-A-R | GAAGCACACGGAATGTTCTA | |
| *NbSc*-B-F | TAGAACATTCCGTGTGCTTC | 1180 |
| *NbSc*-B-R | GTAAAGAGGAATAATGATGG | |
| *NbSc*-C-F | CCATCATTATTCCTCTTTAC | 759 |
| *NbSc*-C-R | GTAACGACAGTGCAACAATG | |
| *NbSc*-D-F | CATTGTTGCACTGTCGTTAC | 808 |
| *NbSc*-D-R | GCCAATGATAGGTCGGGAAT | |
| *NbSc*-E-F | ATTCCCGACCTATCATTGGC | 930 |
| *NbSc*-E-R | CGACCCTCATGCCTTGGAAG | |
| *NbSc*-F-F | CTTCCAAGGCATGAGGGTCG | 546 |
| *NbSc*-F-R | GATCCCATTTTCTTCATTGC | |
| *NbSc*-G-F | GCAATGAAGAAAATGGGATC | 902 |
| *NbSc*-G-R | CAGATCTCGTAGTACATATC | |
| *NbSc*-H-F | CTGGAACATGTTCGACTTCG | 953 |
| *NbSc*-H-R | TGTGCTGGATAACTCGGGCG | |
| *NbSc*-M-F | CCCAACACCCGAACTCAT | - |
| *NbSc*-M-R | AGCCGTCCAACGAAAGCC | |

F: forward primer; R: reverse primer.

**Table S2.** Primers for *N. barkeri* P450s, GSTs and reference genes by *q*PCR.

| Primers | Primer Sequences (5′-3′) |
|---|---|
| *NbCYP4EZ1*-Q-F | ACTACTCACCGATGGATATGCTT |
| *NbCYP4EZ1*-Q-R | TTCGCCACATTGACGTTGTCC |
| *NbCYP4EV2*-Q-F | ATTCTATCGGACACAACGAAC |
| *NbCYP4EV2*-Q-R | CCAGTCAAGAGACCGTTGCC |
| *NbCYP3107E3*-Q-F | CATAAAATATTGCCCCTTCGT |
| *NbCYP3107E3*-Q-R | AAGTTCTTTCGCTTCTCGTCT |
| *NbCYP3110C1*-Q-F | GATGTCATAGCGAACTCGAT |
| *NbCYP3110C1*-Q-R | TCAACTATTTCCTGCCGAAG |
| *NbCYP3110B2*-Q-F | ATGAACCTAAAATACTTGGACCG |
| *NbCYP3110B2*-Q-R | TCTCCTCGTTGTGAATCCTGT |
| *NbCYP3011C2*-Q-F | ATTCCAAAAGGCCAGGTTTGC |
| *NbCYP3011C2*-Q-R | CCATGCCATGATGTCAATGTTCG |
| *NbCYP3011B8*-Q-F | GGTGATATTCTCAAGACCGTTC |
| *NbCYP3011B8*-Q-R | TCACCTCTTTCCATCGCTTC |
| *NbCYP3011B7*-Q-F | ATCTCGTTTCCCGTATTGTGG |
| *NbCYP3011B7*-Q-R | ACAGGATTTGTTCCGTCGTTC |
| *NbCYP3011B10*-Q-F | AGATTTTCACAACTTTGCCGAT |
| *NbCYP3011B10*-Q-R | CAGCCTCTTTGATGTGAACGA |
| *NbCYP3011B9*-Q-F | GCGCTTCCTAATACAACGAT |
| *NbCYP3011B9*-Q-R | GGATCAATGACAGCCCGAAC |
| *NbCYP3103A2*-Q-F | TTCGATAACCCGTATGAGTTCCG |
| *NbCYP3103A2*-Q-R | TTGCCAATTGAGAAGGACCAC |
| *NbCYP3103A3*-Q-F | CTATACTTCGCAACGCTCCT |

**Table S2.** *Cont.*

| Primers | Primer Sequences (5′-3′) |
|---|---|
| *NbCYP3103A3*-Q-R | AGTTTCTGCGATAAGACTCC |
| *NbGSTd01*-Q-F | CTGTTTGAAAGCCGAGCCAT |
| *NbGSTd01*-Q-R | TCCACCTTTGCCCGAGCTTG |
| *NbGSTd02*-Q-F | AATCGAGAGCCATCATGTGC |
| *NbGSTd02*-Q-R | GCTCACAAGTAACGCGAACC |
| *NbGSTd03*-Q-F | AAAGTATTCCGCAAACGACT |
| *NbGSTd03*-Q-R | CCGTTGTATCCGAAATGTCC |
| *NbGSTm03*-Q-F | ATTGAGCTCGGCATAGCACT |
| *NbGSTm03*-Q-R | GACCGCTCCGTCTCTCGAAC |
| *NbGSTo01*-Q-F | CATCCGCCTTTATTGTCACC |
| *NbGSTo01*-Q-R | CCGGTTTATCTTTGACGTTG |
| *NbGSTk02*-Q-F | CCCTGAACACGTCGAAGCAA |
| *NbGSTk02*-Q-R | AGCAACTTCGGGTATGCATT |
| *NbBactin*-Q-F | TACGACCAGAAGCGTACAGC |
| *NbBactin*-Q-R | CCAACCGTGAAAAGATGACC |

F: forward primer; R: reverse primer.
